# Postoperative Weightlifting Restrictions Following Elbow Arthroplasty: A Survey of Italian Society of Shoulder and Elbow Surgery Members

**DOI:** 10.3390/jcm14051577

**Published:** 2025-02-26

**Authors:** Angelo De Crescenzo, Enrico Bellato, Riccardo D’Ambrosi, Gianluca Bullitta, Antonio Benedetto Cecere, Katia Corona, Valentina Fogliata, Gian Mario Micheloni, Maristella Francesca Saccomanno, Fabrizio Vitullo, Andrea Celli, Raffaele Garofalo, SICSeG (Italian Society of Shoulder and Elbow Surgery) Research Committee

**Affiliations:** 1 Shoulder and Elbow Unit, Department of Orthopaedic and Traumatology Surgery, Ente Ecclesiastico Ospedale “F. Miulli”, Acquaviva delle Fonti, 70021 Bari, Italy; raffaelegarofalo@gmail.com; 2 Department of Surgical Science, University of Turin, 10124 Turin, Italy; enrico.bellato@unito.it; 3 San Luigi Gonzaga Hospital, 10043 Orbassano, Italy; 4 IRCCS Ospedale Galeazzi—Sant’Ambrogio, 20157 Milan, Italy; riccardo.dambrosi@hotmail.it; 5 Department of Biomedical Science for Health, University of Milan, 20122 Milan, Italy; 6 CTO Andrea Alesini, 00145 Rome, Italy; bullitta.gianluca@gmail.com; 7 Ospedale San Giuliano, 80014 Giugliano, Italy; antoniobenedettocecere@gmail.com; 8 Department of Medicine and Health Sciences “Vincenzo Tiberio”, University of Molise, 86100 Campobasso, Italy; doc0537@studenti.unimol.it; 9 UO Chirurgia Della Spalla, Cliniche Humanitas Gavazzeni E Castelli, 24125 Bergamo, Italy; valentina.fogliata@gmail.com; 10 Department of Orthopaedic and Traumatology Surgery, University of Modena and Reggio Emilia, 41121 Modena, Italy; gianmario.micheloni@gmail.com; 11 Department of Medical and Surgical Specialties, Radiological Sciences, and Public Health, University of Brescia, 25121 Brescia, Italy; maristellasaccomanno@hotmail.it; 12 Department of Bone and Joint Surgery, Spedali Civili, 25123 Brescia, Italy; 13 Department of Orthopaedic and Traumatology Surgery, University La Sapienza, 00185 Rome, Italy; lino.vitullo@alice.it; 14 Shoulder and Elbow Unit, Department of Orthopaedic Surgery, Hesperia Hospital, 41125 Modena, Italy; celli.andrea.md@gmail.com

**Keywords:** lifting restriction, lifting limitation, elbow arthroplasty, total elbow arthroplasty, survey, postoperative management

## Abstract

**Background:** Total elbow arthroplasty (TEA) has evolved over time from a salvage procedure to a successful treatment of end-stage primary and secondary arthritis. However, the aseptic loosening and the associated reduced survival rate are still concerning. Thus, TEA is typically contraindicated in young and active patients where high-demand activities would promote aseptic loosening. For this reason, postoperative weightlifting limitations are often suggested, yet there is no consensus. The aim of this survey was to collect and analyze the current practice concerning the weightlifting restrictions following elbow arthroplasties among members of the Italian Society of Shoulder and Elbow Surgery (Società Italiana Chirurgia Spalla e Gomito, SICSeG). **Methods:** An online survey on the lifting restrictions after elbow arthroplasties was submitted to all members of the SICSeG. **Results:** In total, 36 members of the Italian society completed the survey. Only five consultants (13.8%) have experience with all the implants analyzed, of whom only three have experience with more than 10 implants per year. Concerning the comprehensive number of elbow arthroplasties performed per year, most of the respondents (45.7%) reported fewer than five surgeries per year, whereas only two surgeons claimed more than 20 procedures. Of the 36 respondents, 32 (88.9%) reported lifelong lifting limitations after linked TEA. In detail, these lifting restrictions were 10 lb in 14 responders (38.9%) and 5 lb in 15 responders (41.7%) performing linked TEA. A lifelong lifting limitation gradually decreased after unlinked TEA and hemiarthroplasty (HA) being advised by 82.8% (24/36) and 64.5% (20/36), respectively. **Conclusions:** To increase implant longevity, most Italian surgeons advise lifting restrictions after TEA. More than 80% of the responders agreed with suggesting lifelong limitations, but a greater variability was found in the amount of weight to which the patients are restricted. Currently, the lack of consensus on the optimal weightlifting restrictions after elbow replacements emphasizes the need for more studies focusing on elbow joint loading during different activities of daily life to improve implant survival rates.

## 1. Introduction

Total elbow arthroplasty (TEA) is an effective and successful surgical procedure performed for a variety of forms of end-stage primary and secondary arthritis, including inflammatory or post-traumatic arthritis, and distal humeral fractures. Recreating a new joint, improved function and reduced pain can be expected with remarkable benefits for quality of life [1]. In addition, elbow instability can be avoided with linked total elbow prosthesis rather than an unlinked design; as an alternative, hemiarthroplasty may represent a valuable solution, especially in cases of acute trauma.

Despite its growing popularity, TEA is still considered an uncommon orthopedic procedure [2]. While the annual number of TEAs performed in the Netherlands was 73 in 2022 [3], the overall number of procedures in the USA over a five-year period was 3146 [4]. After decades of growing incidence, the trends seem to have reached a plateau in recent years [5,6]. However, for the next 40 years, a recent study performed in USA has estimated annual growth of primary and revision TEAs of 1.03% and 5.17%, respectively [5].

The overall survival rate of TEA is of 79% after 11 years, which is notably lower than the 90–95% after 10 years for hip and knee replacements [2,4,7]. This relatively low longevity is mainly the consequence of the concerning overall complication rate of 24% [8]. Considered as the leading cause of implant revision, aseptic loosening is observed in 5.1% of patients with associated symptoms, but the overall incidence of radiolucency is 13.7% [8] (Table 1).

Even though poor cementing technique or osteolysis due to polyethylene debris can play a significant role, the aseptic loosening of TEA must be ascribed to the non-anatomic force transmitted through a linked prosthesis to the bone-cement-implant interface. To address these mechanical issues, several modifications have been introduced over recent decades such as the anterior flange and a less constrained hinge. The floppy hinge of a loosely linked prosthesis allows for about 7–10° of mobility in the varus and valgus direction, but the stability relies more on ligamentous and muscle stabilizers. As observed by Herren et al. [9], varus and valgus laxity in semi-constrained implants can significantly increase from 9.6° when ligaments are repaired to 12.8° with collateral ligaments removed. As a matter of fact, the ligamentous and muscle stabilizers are not addressed during the TEA procedure and potential column removal and/or resorption would weaken these stabilizers. In addition, radial head excision can increase valgus-varus laxity when performed, as shown by King et al. in unconstrained implants [10]. In most unconstrained prostheses, the radiocapitellar joint is not addressed and the radial head is resected. In this way, up to 60% of loads, especially between 0° and 30° of elbow flexion, are transferred to the new hinge [11]. Then, as explained by Wolff’s law, significant bone resorption is accordingly observed in the condylar bone [7]. These factors subsequently increase the lever arm between the hinge and the diaphyseal stem fixation and ultimately the risk of several complications, such as polyethylene wear, stem loosening, mechanical failure, or periprosthetic fracture.

Bearing in mind these inherent biomechanical drawbacks, TEA is typically contraindicated in young and active patients where high-demand activities would promote aseptic loosening. Nonetheless, there is no consensus on activity restrictions and whether these should be mandatory following linked TEA to minimize the risk of implant loosening and increase longevity. For instance, contradictions can be found among the manufacturers of the most implanted TEA; some recommend a lifelong lifting limitation of 5 lb (Coonrad-Morrey and Nexel arthroplasties from Zimmer Biomet, Warsaw, Indiana) [12] while others do not advise any specific weight restrictions (Discovery system from LimaCorporate, San Daniele del Friuli, Italy and Latitude system from Wright-Tornier, Memphis, Tennessee) [13,14]. Similarly, no guidelines are specified even after unlinked TEA or hemiarthroplasty (HA), which are less constrained implants.

The aim of this survey was to analyze the current practice of Italian surgeons with their postoperative indications, with specific attention to the activities and lifting limitations recommended. To that end, a questionnaire-based survey was submitted to specialized orthopedic surgeons, and members of the Italian Society of Shoulder and Elbow Surgery (Società Italiana Chirurgia Spalla e Gomito, SICSeG).

## 2. Materials and Methods

An online survey on lifting restrictions after hemiarthroplasty (HA) and TEA (both linked and unliked) was submitted to all 262 members of the Italian Society of Shoulder and Elbow Surgery (Società Italiana Chirurgia Spalla e Gomito, SICSeG) through email and using the web-based survey tool “google forms” (134.0.6998.23/19 February 2025, Mountain View, CA, USA). The questionnaire was created and validated by the SICSeG “Research Committee” and disseminated by SICSeG secretary’s office to all members at the beginning of April 2024. Then, at the national meeting held from 11 to 13 April, a reminder was issued to all participants and then the survey was closed 4 weeks later.

The survey consisted of 17 questions about: general information on the respondents’ experience; postoperative physiotherapy; recommendations for immobilization and specific instructions concerning lifelong activities after linked TEA, unlinked TEA, and HA; and any open insights into weightlifting restrictions and the frequency of their follow-up.

With the first three questions, the survey aimed to classify the respondents according to their surgical activities and experience, in terms of the number of overall implants performed per year, the type of arthroplasty most frequently performed, and the pathology treated. Then, the respondents were asked whether they had a rehabilitation protocol in their hospital and whether physiotherapists were involved in the inpatients’ postoperative phase. As the major section of the survey, the following ten questions examined the postoperative indications and limitations recommended for each type of elbow replacement. Two of these evaluated for how long motion was restricted after HA and TEA (no immobilization, 1, 2, 3, 4, and 6 weeks of immobilization). In the following eight questions, all the respondents were asked whether they advised lifting limitations for each type of implant, for how long this was recommended (6 weeks, 12 weeks, lifelong, or never), and the amount of weight advised (2, 5, 10, and 20 pounds). The remaining two out of the ten main sections asked whether the respondents suggested limitations in sport activities after HA and TEA. In conclusion, the last two questions asked whether each respondent could offer any insights into the importance of weightlifting and activity restrictions, and then the follow-up frequency the respondents usually used in their practice after the first year after the surgery (each year, each 2 years, never after the first year).

## 3. Results

In total, 36 members of the Italian society completed the survey. All the respondents were consultants, and none were residents or fellows. Each question was answered by any responder.

In the first section, evaluating the numbers and types of implant replacement performed in one year, the most implanted arthroplasty is linked TEA, which was performed by 34 surgeons (94.4%). Only five consultants (13.8%) have experience with all the implants analyzed, of whom only three have experience with more than 10 implants per year. Regarding the comprehensive number of elbow arthroplasties performed per year, most of the respondents (45.7%) reported fewer than five surgeries per year, whereas only two surgeons claimed more than 20 procedures.

**Figure 1:**
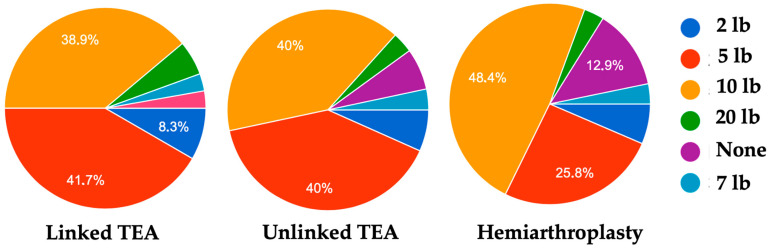
Comparison among the amount of weight restrictions after linked TEA, unlinked TEA, and hemiarthroplasty.

For early postoperative management, 58.3% (21/36) of the respondents asserted the absence of any rehabilitation protocol in their hospital, whereas 27.8% (10/36) routinely use a rehabilitation protocol for both HA and TEA (Table 1). Conversely, physiotherapists are employed in 60% of the surveyed surgeons’ hospitals (45.7% for both HA and TEA, and only 14.3% for TEA) (Table 2). Physiotherapy after TEA begins the day after surgery for most of the interviewed surgeons (19/36, 52.8%), but 27.8% (10/36) advise immobilization for the first two weeks (Table 3). The situation is somehow different after an HA, where as much as 43.8% (14/36) of the respondents choose a more conservative approach with an immobilization for two weeks (Table 3). Of the 36 respondents to the survey, 32 (88.9%) of them advise lifelong lifting limitations after linked TEA. In detail, these lifting restrictions were of 10 lb for 14 responders (38.9%) and 5 lb for 15 responders (41.7%) performing linked TEA (Table 4). The lifelong lifting limitation gradually decreased after unlinked TEA and HA, being advised by 82.8% (24/36) and 64.5% (20/36) of the respondents, respectively. Lifting limitations of 5 and 10 lb were recommended by 40% for each group after unlinked TEA, whereas after the HA 10 lb was advised by a greater number of surgeons, that is, 48.4% (15 respondents) (Table 4, Figure 1).

## 4. Discussion

This survey shows a substantial agreement among Italian surgeons concerning the importance of lifting restrictions after TEA but a significant variability in terms of the amount of the weight recommended. More than 80% of the responders suggest lifelong lifting limitations after either linked or unliked TEA. Conversely, the amount of weight advised is more unpredictable. After a linked implant, 5 lb and 10 lb were advised by 43.7% and 37.5% of surgeons, respectively. Similarly, the same restrictions were suggested by 46% and 39% of the respective responders performing unlinked TEA. As a result, 11.1% of surgeons after linked TEA and 17.2% of surgeons surveyed after unlinked TEA do not advise any lifelong weight restriction.

This survey shows different results from a similar study performed among the British Elbow and Shoulder Society (BESS) members [15]. British colleagues advising lifelong lifting limitation were fewer than those of this survey, with a rate of 78% and 61% following linked and unlinked TEA, respectively [15]. However, a similar trend towards higher lifting limitations was observed after an unlinked prosthesis [15]. If 5 lb represents the most suggested restriction after linked TEA, a greater rate of responders advising 10 lb was found after an unlinked implant. With an unlinked implant, a lower force is transmitted through the humeral and ulnar stems, allowing greater postoperative activities than a linked prosthesis [1]. Thus, the lower constraint within unlinked TEA can explain the increased amount of weight usually observed.

Conversely, the replies collected by the Italian surgeons in this survey were more similar to those described by van Dam et al., who conducted an interview among European orthopedic surgeons members of the European Society for Surgery of the Shoulder and the Elbow (SECEC/ESSSE) [16]. Of the 54 respondents, the majority (49/54, 91%) recommended weight-bearing restrictions after TEA. In detail, 30 out of 41 respondents (73%) advised 1–5 kg, six respondents (15%) advised less than 1 kg, and four respondents (10%) advised 1–10 kg [16].

Of the surveyed surgeons performing HA, interestingly, 64.5% suggest lifelong lifting limitations. Although lower than after unlinked TEA, this rate is consistently different from those of previous studies. In the British survey, 68% of the consultants did not suggest any lifelong lifting limitations after HA [15]. These less frequent restrictions are supported by a different implant design, where the joint stability relies on the healing of collateral ligaments to the implant. Aseptic loosening is a rarer indication for revision after HA than TEA [1], but joint instability is conversely one of the most common effects. For this reason, understanding the consequences of lifting and heavy activities on proper collateral ligaments healing remains crucial to guide rehabilitation and maximize implant longevity. For these reasons, a different postoperative rehabilitation program can be observed among the responders. If most of the responders (52.8%) allow joint motion from the day after the surgery for TEA, the majority of them prefer a rest period of 15 days after surgery for the HA (Table 3).

This survey shows a small number of procedures performed by most of the responders. In fact, most of the respondents (45.7%) reported fewer than five surgeries per year whereas only two surgeons perform more than 20 procedures per year. Considering the relatively high rate of postoperative complications and the low rate of agreement in the postoperative recommendations, this aspect should be analyzed and addressed.

The unclear postoperative recommendations are supported by the manufacturers of the four most implanted TEA, which give different advice even though they are all linked and semi-constrained implants. While for the Coonrad-Morrey and Nexel (Zimmer Biomet, Warsaw, Indiana) they recommend a lifelong lifting limitation of 5 lb [12], those of Discovery (LimaCorporate, San Daniele del Friuli, Italy) and Latitude (Wright-Tornier, Memphis, Tennessee) do not advise any specific weight restrictions, but only to start strengthening exercises after 6 and 10 weeks from surgery, respectively [13,14].

As a matter of fact, the weightlifting restriction is likely a simplistic way to reduce implant wear and loosening. Through the analysis of a retrieved TEA, polyethylene wear consistent with a varus and valgus load has been demonstrated [17]. Lo and Lipman observed that 5 Nm varus-valgus moments would exceed the polyethylene strength of the Coonrad-Morrey prosthesis, leading to irreversible deformation [18]. King et al. have shown polyethylene deformation in three different types of TEAs with a 2.3-kg weight when the shoulder was abducted at 45° and 90° [19]. Besides varus-valgus moments, biomechanical studies have shown greater energies transmitted through the radiocapitellar joint within the first 30° of flexion and with pronated forearm [20,21]. As observed by Kincaid and An, the peak bone-on-bone contact forces occur in an almost fully extended elbow position, that is, between 7° and 11° of flexion [20]. This could be the consequence of the poor mechanical advantage of the prime elbow movers in full extension, which are the brachialis, biceps, and brachioradialis muscles [22].

Thus, the shoulder, elbow, and hand positions in the space play a relevant role in determining the load transferred through the elbow. This would determine the joint moments in flexion-extension, pronation-supination, and varus-valgus movement directions. Duijn et al. have recently investigated elbow joint moments when performing eight common daily tasks [4]. The most demanding activities with the highest peak moments were observed when rising from a chair (13.4 Nm extension, 5.0 Nm supination, and 15.2 Nm varus), followed by steering a car (9.3 Nm extension, 5.4 Nm supination, and 15.2 Nm valgus) [4]. Conversely, sliding an object required the smallest elbow moment (4.3 Nm flexion, 1.7 Nm supination, and 2.6 Nm valgus) [4]. However, they found that the peak flexion-extension and varus-valgus moments were higher than the peak of pronation-supination moments [4]. Moreover, greater moments were detected, in all directions, when an opposing external force was applied to the hand. Rising from a chair and steering a car were the two most demanding activities, likely due to the combination of large external forces and large moment arm [4]. In addition, the authors interestingly examined whether the instruction of “not lifting more than 1 kg” would lead to a decreased joint moment. As expected, the instructions did not have any significant effect, except for the task “lifting from a chair”. The authors hypothesized that this was likely the consequence of the combination of the other arm and leg movements helping while lifting their own weight [4].

Besides the amount of weight and the joint moments, task frequency also plays a role in determining the final load to the prosthesis. Many repetitions of the same task will lead to a potential greater polyethylene load and ultimately a higher risk of aseptic loosening. It is therefore important to also bear in mind the frequency of the task.

In conclusion, the results achieved by biomechanical studies are not generalizable to daily activities and the restriction referred to a specific weight may not be enough reliable. To maximize implant longevity, lifelong weight restrictions are a crucial point, but postoperative limitations should probably also focus on reducing those joint moments which are more unsafe, in the flexion-extension and varus-valgus directions. A description of activities to avoid rather than recommendations on weight restrictions would be better received by patients. In fact, patient’s compliance with postoperative recommendations can be crucial. This aspect was emphasized by a survey performed in the USA by Barlow and colleagues, during which they interviewed 113 patients following TEA [23]. Despite 80% of these remembering having received postoperative restrictions, the authors interestingly observed 40% of patients performing high-demand activities, such as snow shoveling, wheelbarrow use, hunting, and shooting [23]. To further confirm the poor understanding of postoperative recommendations, 83% of the responders believed they were compliant with these restrictions [23]. The lowest compliance with the physician’s indications were observed in those undergoing surgery for acute fracture or fracture nonunion [23].

As with any survey, the results of this study may be skewed by inherent bias. As it was an online and voluntary interview, the survey could not receive replies from every member of the SICSeG. The questionnaire focused on primary TEA and HA, and not on revision TEA. This represents a more complex scenario, with more factors to be considered for postoperative management. The results achieved by the questionnaire are not linked to patients’ outcomes, which would improve the value of the conclusions. In addition, surgeons who were not members of the Italian Society at the time of the survey were not interviewed. The survey was sent to 262 consultant members of the SICSeG, and replies were received from 36 surgeons (14%). As the SICSeG does not split its members into shoulder and/or elbow specialists, it is not possible to precisely identify the experienced surgeons. In addition, the lack of a national registry makes the selection criteria further complex. Considering the niche surgery with which the survey deals and the limited number of specialized centers performing these procedures, we believe that the number of surveyed surgeons analyzed may be representative of the Italian surgical attitude. However, the rate of responders is similar to that of previous surveys [15,16]. A future aim for international elbow societies or communities could be to aggregate a larger number of surgeons’ opinions to improve the validity of these questionnaires.

## 5. Conclusions

To increase implant longevity, most Italian surgeons advise lifting restrictions after TEA. Even though more than 80% of the responders agree with suggesting lifelong limitations, a greater variability was found in the amount of weight to which the patients are restricted. Interestingly, most of the surveyed surgeons support lifelong weight limitations even after an HA. This great variability emphasizes the lack of consensus on the optimal weightlifting restrictions after elbow replacements. Thus, more studies and, specifically, biomechanical studies focusing on elbow joint loading during different activities of daily living are warranted to improve implant survival rates.

## Figures and Tables

**Table 1:** Complications of total elbow arthroplasties [8].

Complication	Incidence (%)
Aseptic loosening (clinical)	5.1 ± 3.4
Aseptic loosening (clinical and radiographic)	
Linked designs	13.7 ± 6.8
Unlinked designs	10.1 ± 4.8
Dislocation/subluxation	4.7 ± 3.0
Infections, deep	3.3 ± 2.9
Intraoperative fractures	3.0 ± 2.7
Fractures of prosthesis	2.9 ± 3.6
Ulnar nerve complications	2.9 ± 2.4
Delayed healing	2.5 ± 2.6
Postoperative fractures	2.4 ± 2.1
Triceps complications	2.4 ± 2.4
Bushing wear	2.3 ± 3.4
Disassembly	2.3 ± 3.5

**Table 2:** Postoperative physiotherapy management following elbow arthroplasty?

	Does Your Department Use a Postoperative Rehabilitation Program?	Are Physiotherapists Routinely Involved in Your Inpatient Rehabilitation Program?
	% (*n*)	% (*n*)
Yes, for both total elbow arthroplasty and hemiarthroplasty	27.8% (10/36)	45.7% (16/35)
Yes, for total elbow arthroplasty	13.9% (5/36)	14.3% (5/35)
Yes, for hemiarthroplasty	0% (0/36)	0% (0/35)
No	58.3% (21/36)	40% (14/35)
Not sure	0% (0/36)	0% (0/35)

**Table 3:** Joint immobilization following elbow arthroplasty?

In Your Postoperative Rehabilitation Program, When Do You Allow Joint Motion?		
	Total Elbow Arthroplasty	Hemiarthroplasty
	% (*n*)	% (*n*)
The day after the surgery	52.8% (19/36)	31.3% (10/32)
After 7 days	11.1% (4/36)	15.6% (5/32)
After 15 days	27.8% (10/36)	43.8% (14/32)
After 21 days	5.6% (2/36)	3.1% (1/32)
After 1 month	2.8% (1/36)	6.3% (2/32)
After 45 days	0% (0/36)	0% (0/36)

**Table 4:** Weightlifting restriction following elbow arthroplasty?

	Linked Total Elbow Arthroplasty	Unlinked Total Elbow Arthroplasty	Hemiarthroplasty
Do You Recommend Weightlifting Restrictions Post-Operatively?			
	% (*n*)	% (*n*)	% (*n*)
Yes, for the first 6 weeks	0% (0/36)	0% (0/29)	9.7% (3/31)
Yes, for the first 12 weeks	11.1% (4/36)	10.3% (3/29)	16.1% (5/31)
Yes, lifelong	88.9% (32/36)	82.8% (24/29)	64.5% (20/31)
No	0% (0/36)	6.9% (2/29)	9.7% (3/31)
**If You Recommend Weight Limitations, What Weight Do You Allow Them to Lift?**			
	**% (*n*)**	**% (*n*)**	**% (*n*)**
2 pounds (~1 kg)	8.3% (3/36)	6.7% (2/30)	6.5% (2/31)
5 pounds (~2.25 kg)	41.7% (15/36)	40% (12/30)	25.8% (8/31)
10 pounds (~4.5 kg)	38.9% (14/36)	40% (12/30)	48.4% (15/31)
20 pounds (~9 kg)	5.6% (2/36)	3.3% (1/30)	3.2% (1/31)
No lifelong lifting limitations	0% (0/36)	6.7% (2/30)	12.9% (4/31)
Other	5.6% (2/36)	3.3% (1/30)	3.2% (1/31)

## Data Availability

Dataset available on request from the authors.
